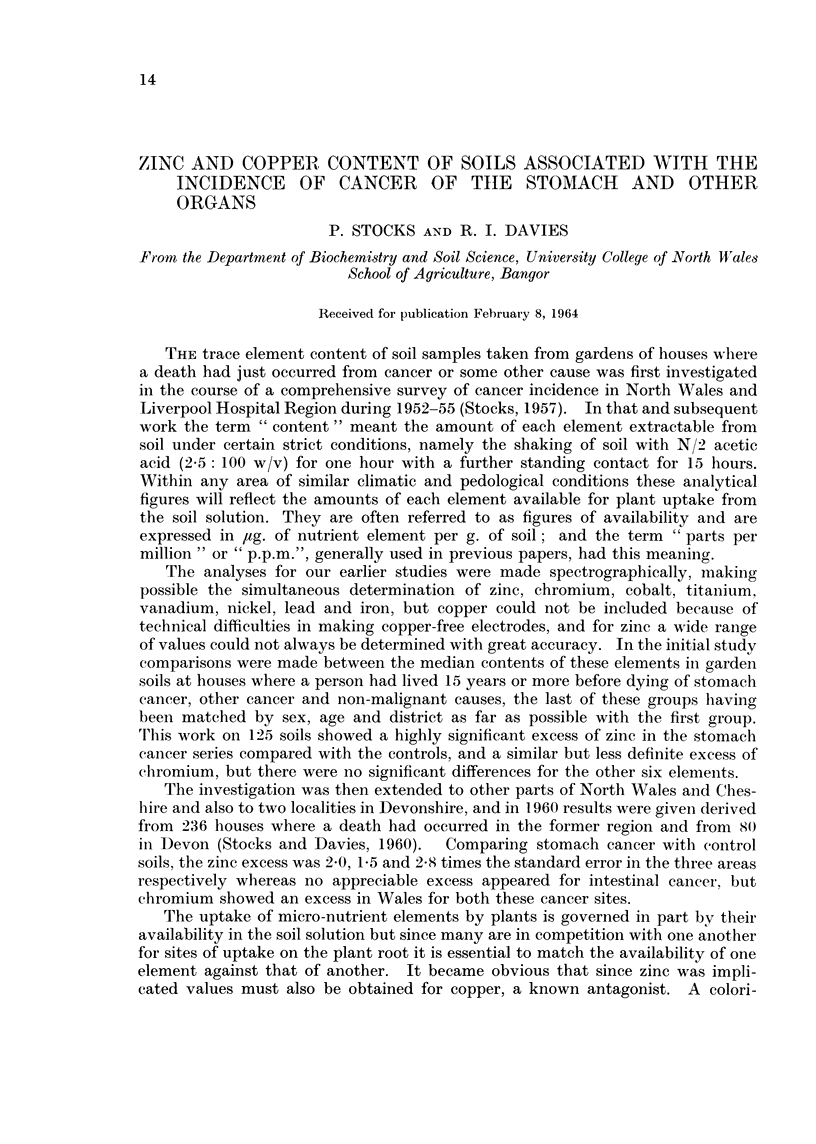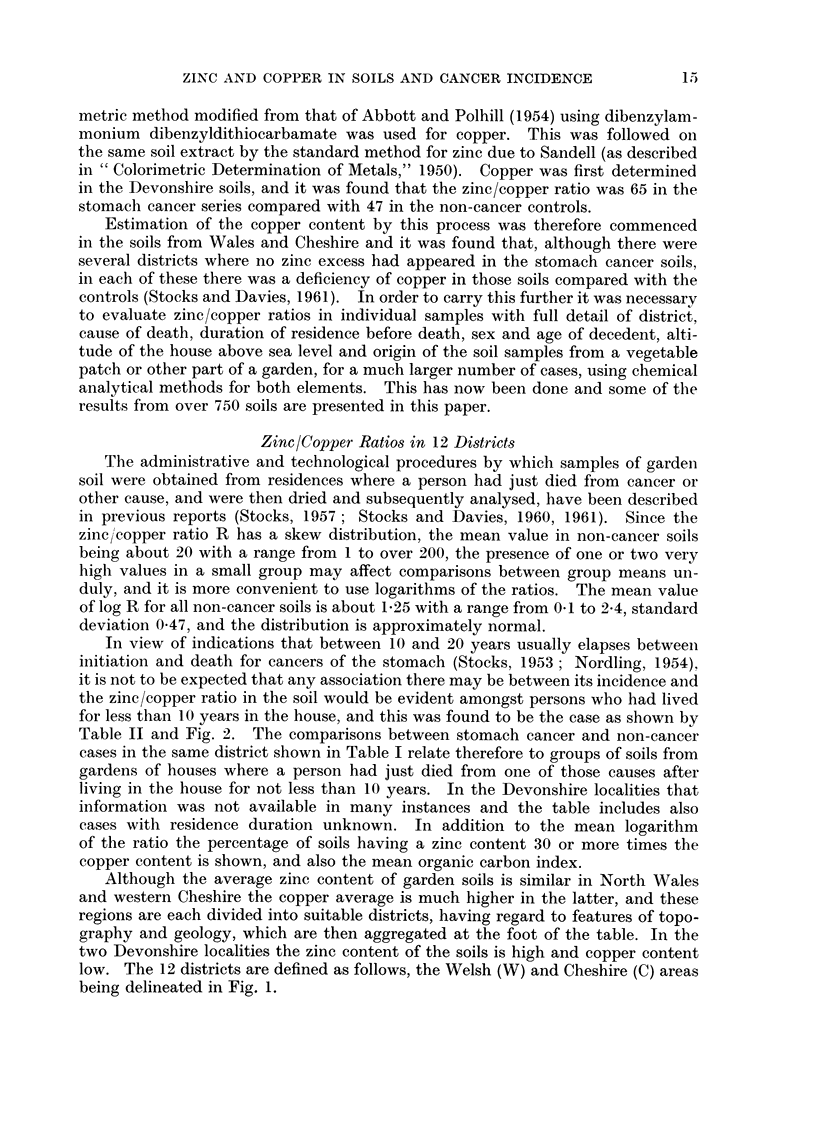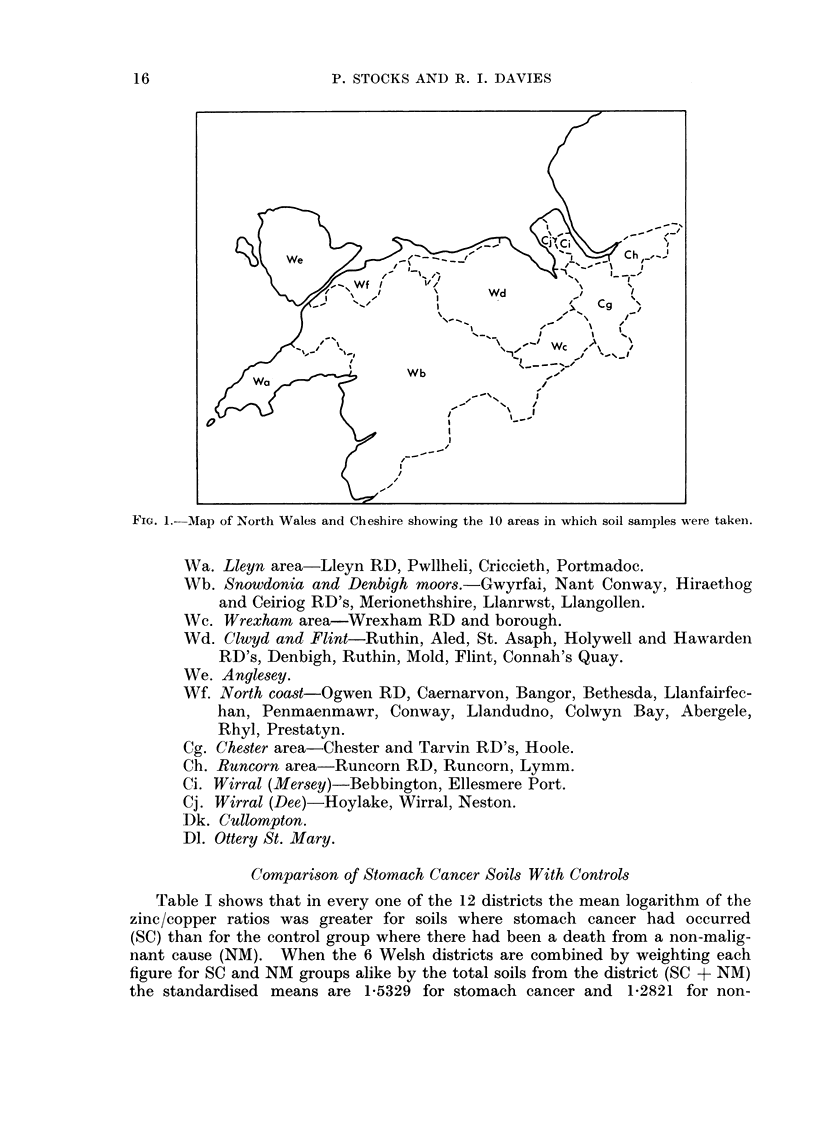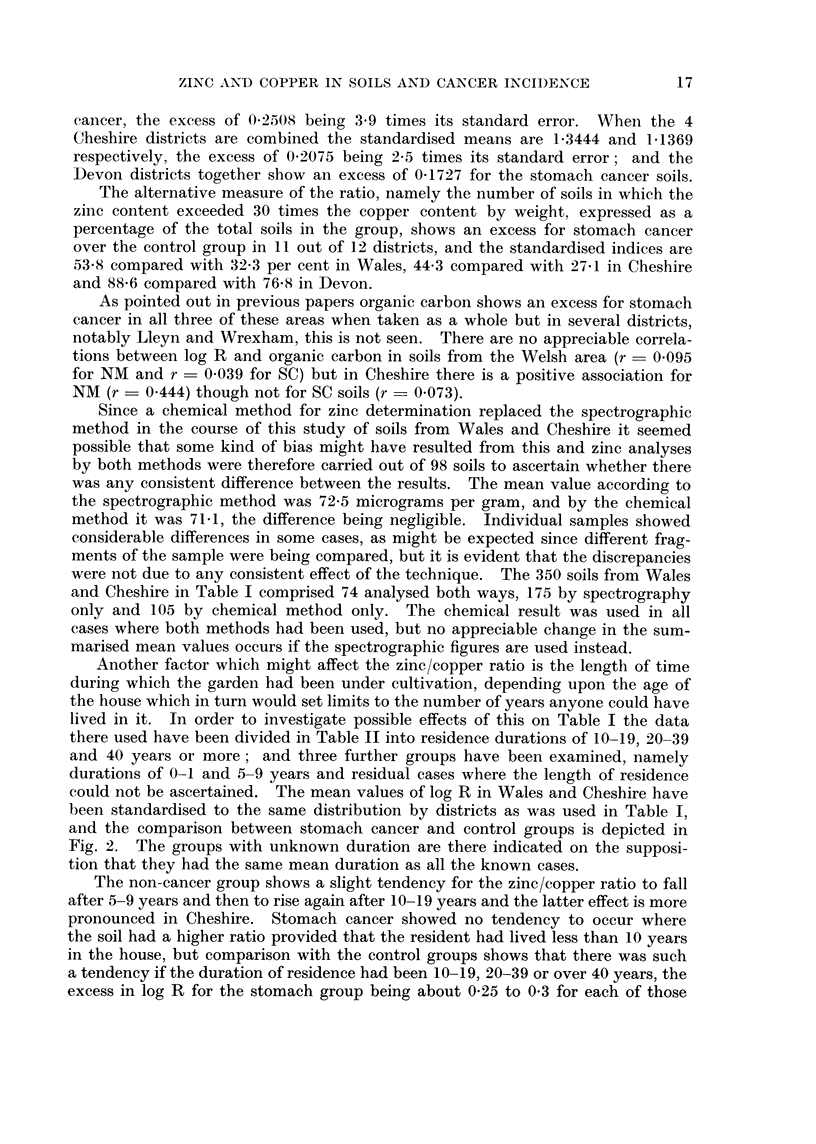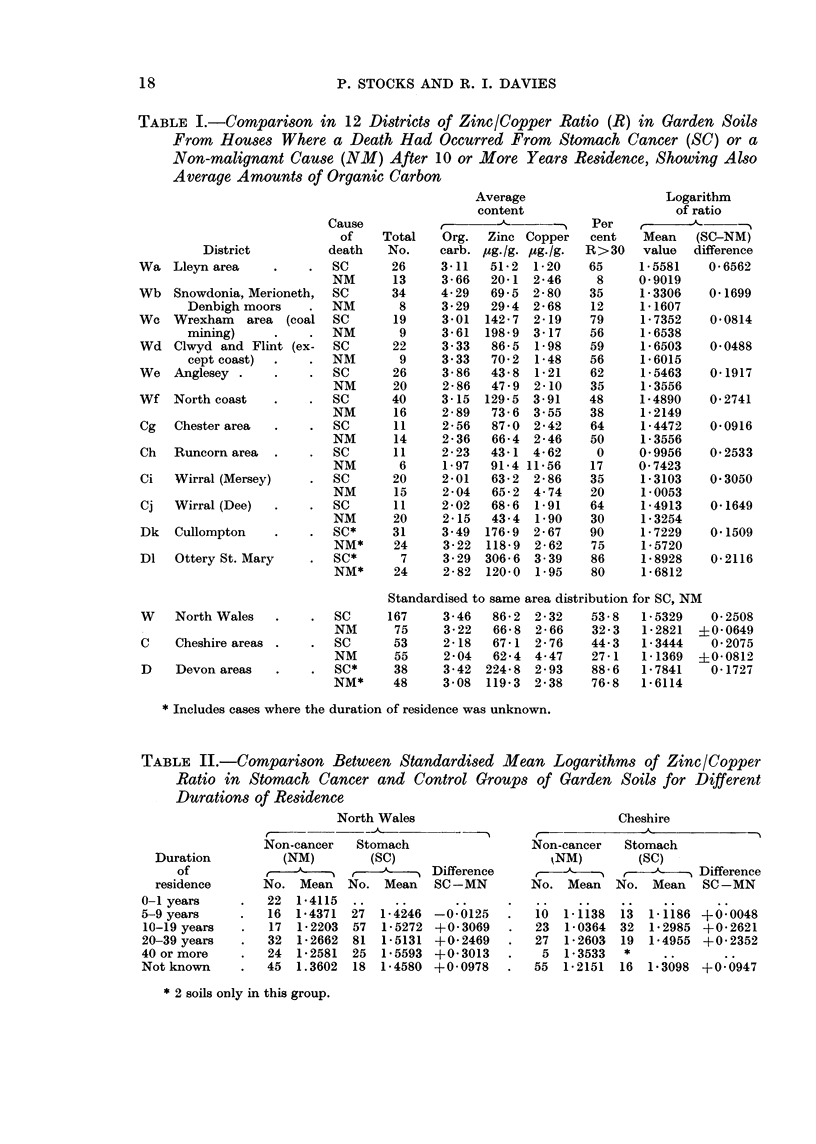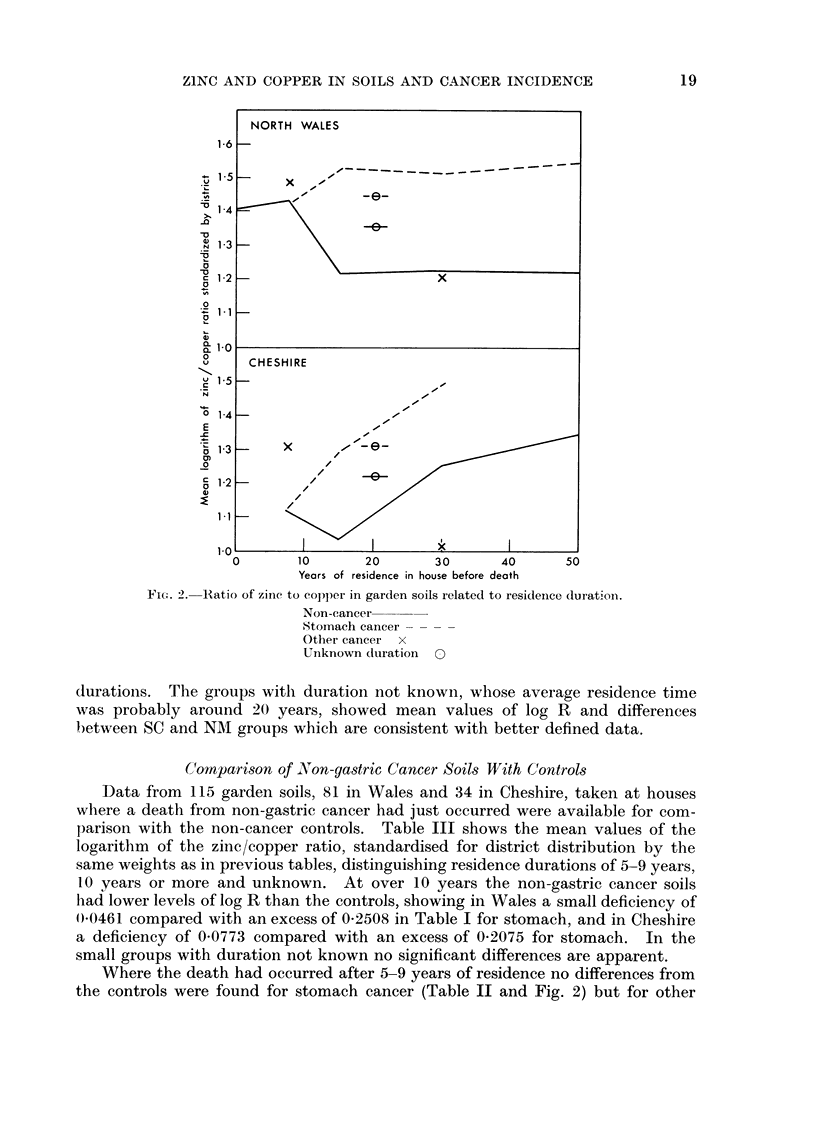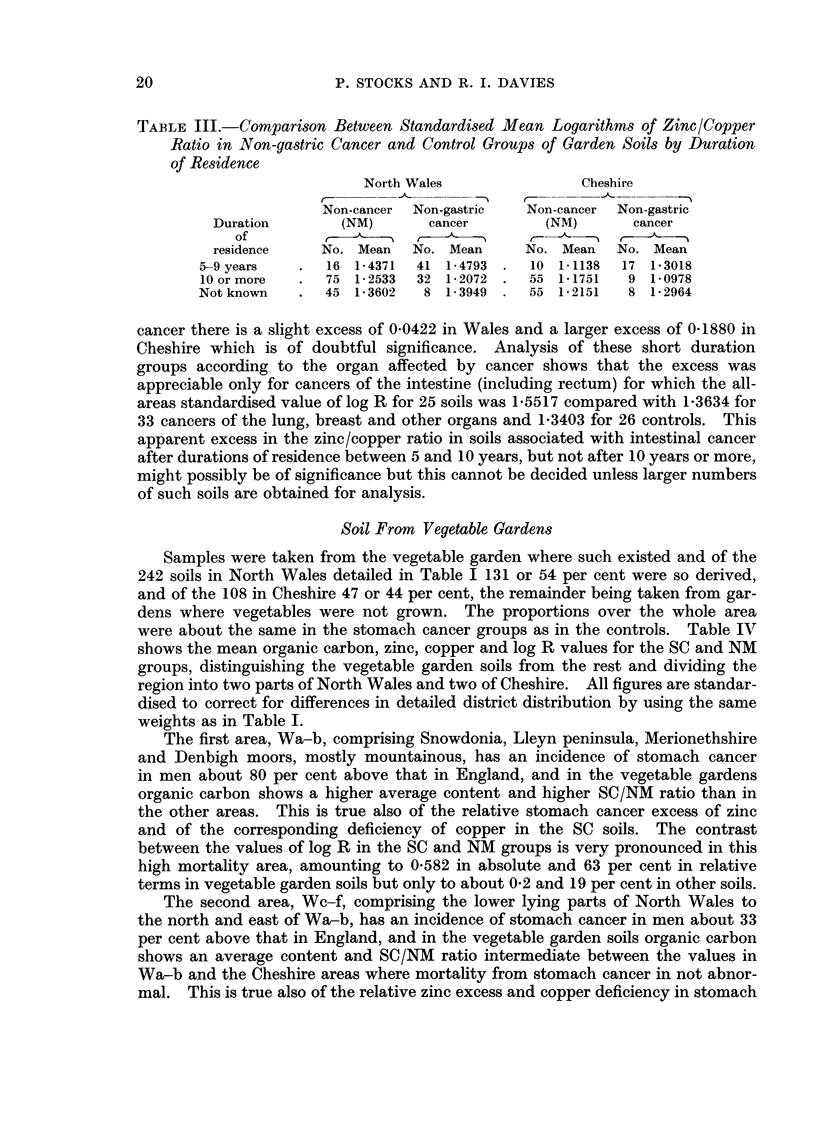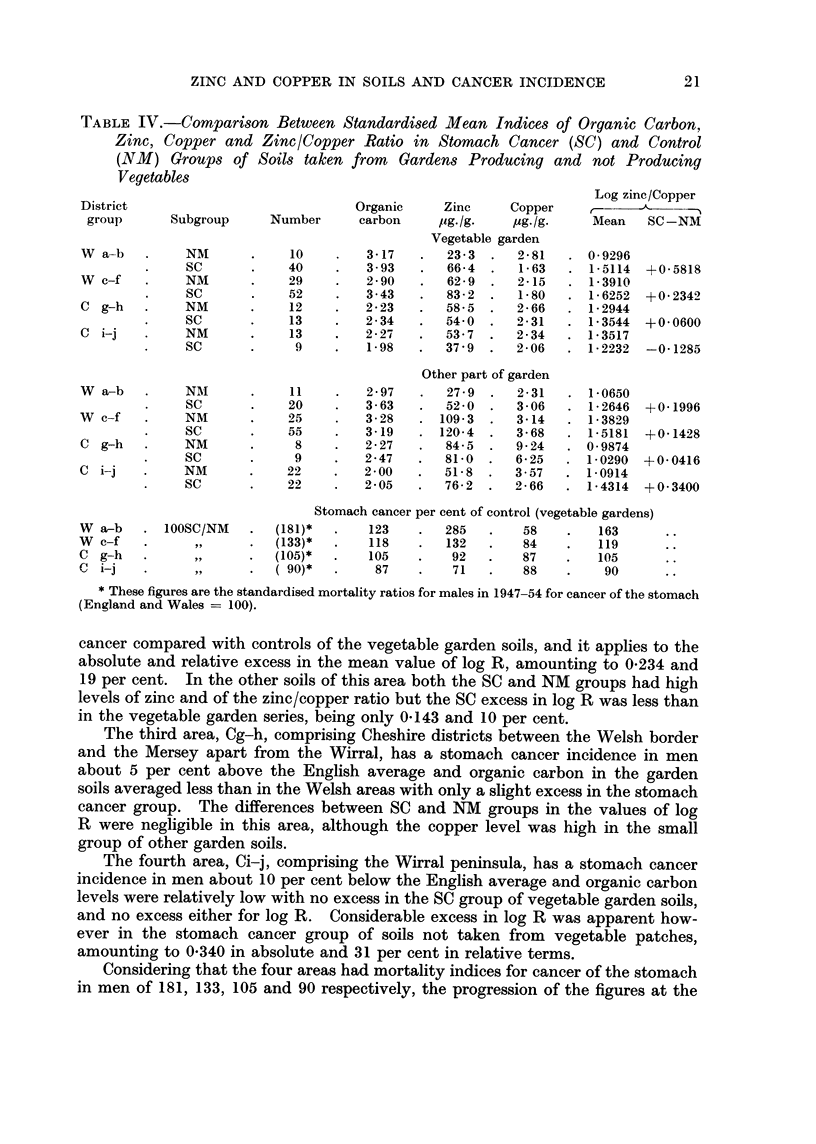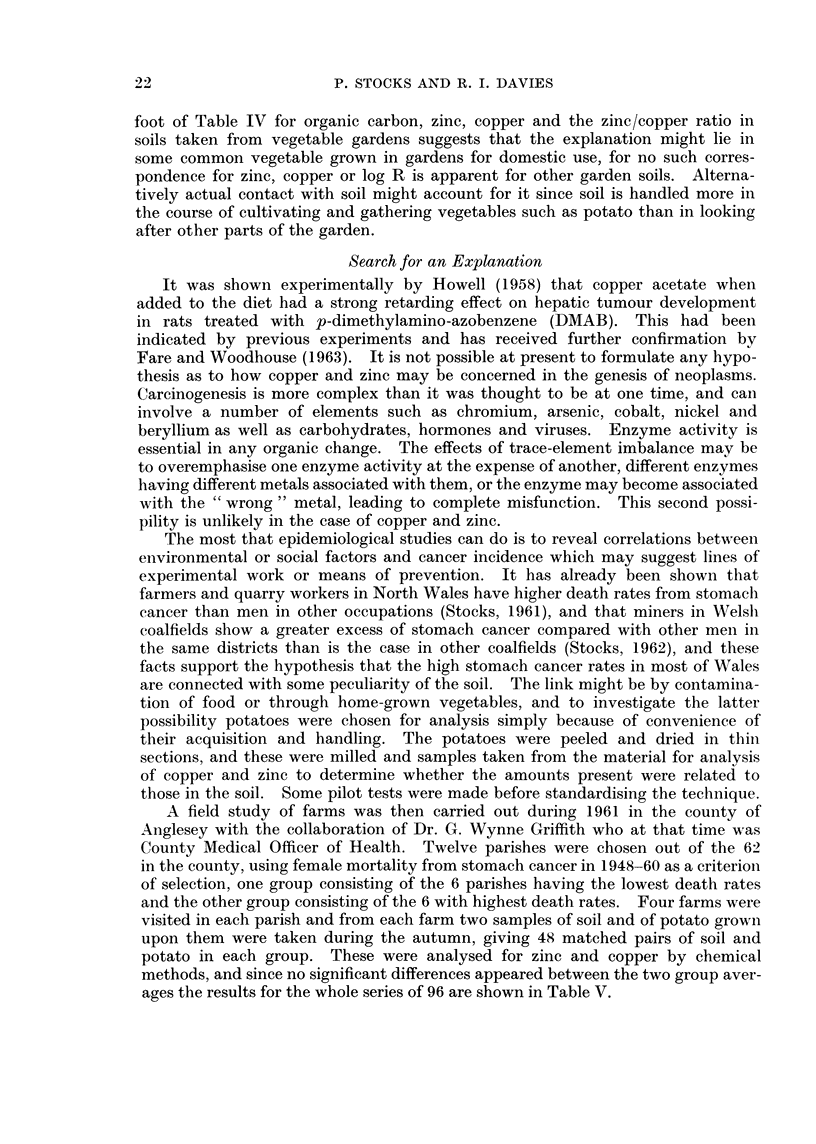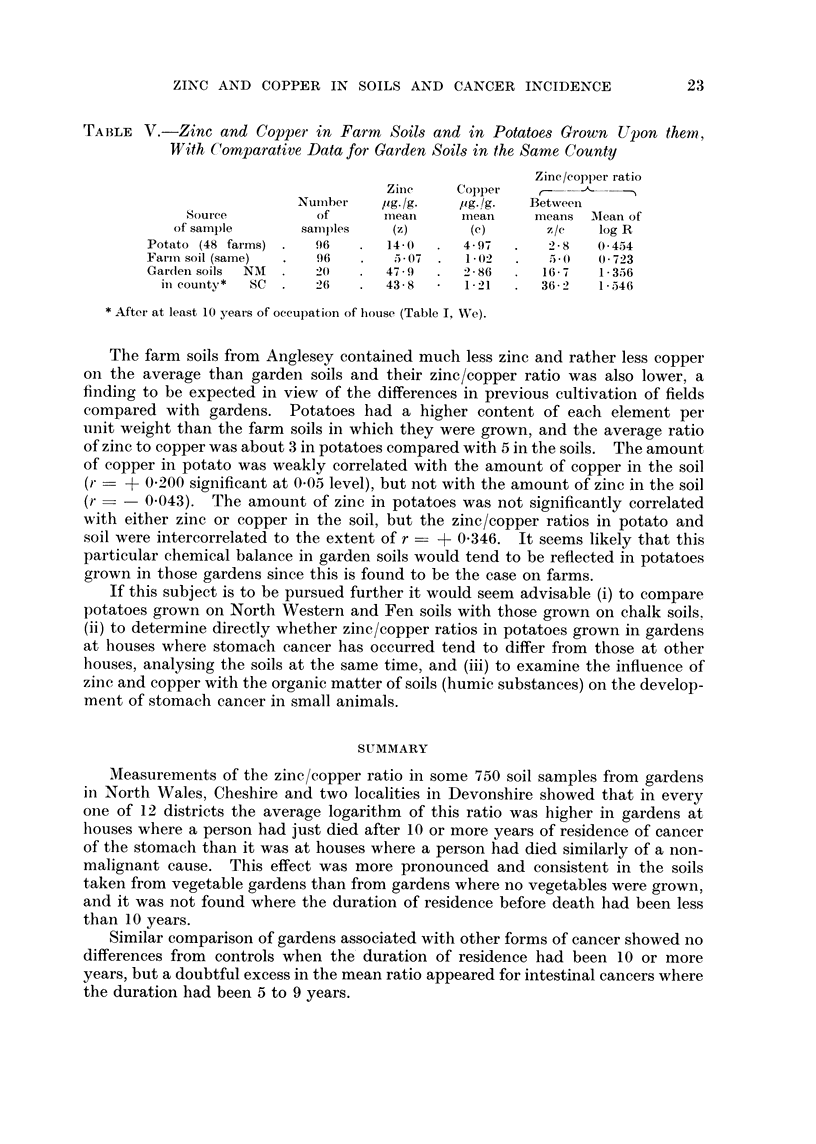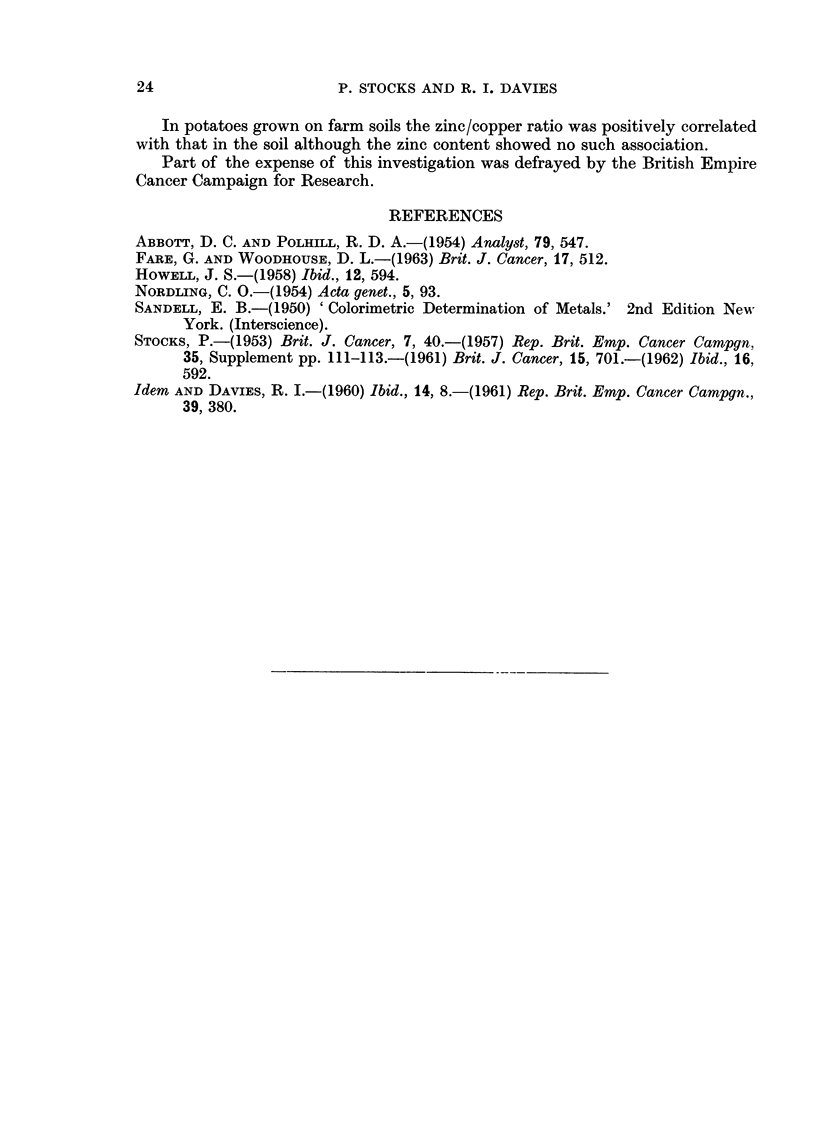# Zinc and Copper Content of Soils Associated with the Incidence of Cancer of the Stomach and other Organs

**DOI:** 10.1038/bjc.1964.2

**Published:** 1964-03

**Authors:** P. Stocks, R. I. Davies


### ZINC AND COPPER CONTENT OF SOILS ASSOCIATED WITH THE INCIDENCE OF CANCER OF THE STOMACH AND OTHER ORGANS

P. STOCKS AND R. I. DAVIES

*From the Department of Biochemistry and Soil Science, University College of North Wales School of Agriculture, Bangor*

Received for publication February 8, 1964

THE trace element content of soil samples taken from gardens of houses where a death had just occurred from cancer or some other cause was first investigated in the course of a comprehensive survey of cancer incidence in North Wales and Liverpool Hospital Region during 1952–55 (Stocks, 1957). In that and subsequent work the term " content " meant the amount of each element extractable from soil under certain strict conditions, namely the shaking of soil with N/2 acetic acid (2·5 : 100 w/v) for one hour with a further standing contact for 15 hours. Within any area of similar climatic and pedological conditions these analytical figures will reflect the amounts of each element available for plant uptake from the soil solution. They are often referred to as figures of availability and are expressed in $\mu$g. of nutrient element per g. of soil ; and the term " parts per million " or " p.p.m.", generally used in previous papers, had this meaning.

The analyses for our earlier studies were made spectrographically, making possible the simultaneous determination of zinc, chromium, cobalt, titanium, vanadium, nickel, lead and iron, but copper could not be included because of technical difficulties in making copper-free electrodes, and for zinc a wide range of values could not always be determined with great accuracy. In the initial study comparisons were made between the median contents of these elements in garden soils at houses where a person had lived 15 years or more before dying of stomach cancer, other cancer and non-malignant causes, the last of these groups having been matched by sex, age and district as far as possible with the first group. This work on 125 soils showed a highly significant excess of zinc in the stomach cancer series compared with the controls, and a similar but less definite excess of chromium, but there were no significant differences for the other six elements.

The investigation was then extended to other parts of North Wales and Cheshire and also to two localities in Devonshire, and in 1960 results were given derived from 236 houses where a death had occurred in the former region and from 80 in Devon (Stocks and Davies, 1960). Comparing stomach cancer with control soils, the zinc excess was 2·0, 1·5 and 2·8 times the standard error in the three areas respectively whereas no appreciable excess appeared for intestinal cancer, but chromium showed an excess in Wales for both these cancer sites.

The uptake of micro-nutrient elements by plants is governed in part by their availability in the soil solution but since many are in competition with one another for sites of uptake on the plant root it is essential to match the availability of one element against that of another. It became obvious that since zinc was implicated values must also be obtained for copper, a known antagonist. A colorimetric method modified from that of Abbott and Polhill (1954) using dibenzylammonium dibenzyldithiocarbamate was used for copper. This was followed on the same soil extract by the standard method for zinc due to Sandell (as described in " Colorimetric Determination of Metals," 1950). Copper was first determined in the Devonshire soils, and it was found that the zinc/copper ratio was 65 in the stomach cancer series compared with 47 in the non-cancer controls.

Estimation of the copper content by this process was therefore commenced in the soils from Wales and Cheshire and it was found that, although there were several districts where no zinc excess had appeared in the stomach cancer soils, in each of these there was a deficiency of copper in those soils compared with the controls (Stocks and Davies, 1961). In order to carry this further it was necessary to evaluate zinc/copper ratios in individual samples with full detail of district, cause of death, duration of residence before death, sex and age of decedent, altitude of the house above sea level and origin of the soil samples from a vegetable patch or other part of a garden, for a much larger number of cases, using chemical analytical methods for both elements. This has now been done and some of the results from over 750 soils are presented in this paper.

*Zinc/Copper Ratios in 12 Districts*

The administrative and technological procedures by which samples of garden soil were obtained from residences where a person had just died from cancer or other cause, and were then dried and subsequently analysed, have been described in previous reports (Stocks, 1957; Stocks and Davies, 1960, 1961). Since the zinc/copper ratio R has a skew distribution, the mean value in non-cancer soils being about 20 with a range from 1 to over 200, the presence of one or two very high values in a small group may affect comparisons between group means unduly, and it is more convenient to use logarithms of the ratios. The mean value of log R for all non-cancer soils is about 1·25 with a range from 0·1 to 2·4, standard deviation 0·47, and the distribution is approximately normal.

In view of indications that between 10 and 20 years usually elapses between initiation and death for cancers of the stomach (Stocks, 1953; Nordling, 1954), it is not to be expected that any association there may be between its incidence and the zinc/copper ratio in the soil would be evident amongst persons who had lived for less than 10 years in the house, and this was found to be the case as shown by Table II and Fig. 2. The comparisons between stomach cancer and non-cancer cases in the same district shown in Table I relate therefore to groups of soils from gardens of houses where a person had just died from one of those causes after living in the house for not less than 10 years. In the Devonshire localities that information was not available in many instances and the table includes also cases with residence duration unknown. In addition to the mean logarithm of the ratio the percentage of soils having a zinc content 30 or more times the copper content is shown, and also the mean organic carbon index.

Although the average zinc content of garden soils is similar in North Wales and western Cheshire the copper average is much higher in the latter, and these regions are each divided into suitable districts, having regard to features of topography and geology, which are then aggregated at the foot of the table. In the two Devonshire localities the zinc content of the soils is high and copper content low. The 12 districts are defined as follows, the Welsh (W) and Cheshire (C) areas being delineated in Fig. 1.

FIG. 1.—Map of North Wales and Cheshire showing the 10 areas in which soil samples were taken.

Wa. *Lleyn* area—Lleyn RD, Pwllheli, Criccieth, Portmadoc.
Wb. *Snowdonia and Denbigh moors.*—Gwyrfai, Nant Conway, Hiraethog and Ceiriog RD's, Merionethshire, Llanrwst, Llangollen.
Wc. *Wrexham* area—Wrexham RD and borough.
Wd. *Clwyd and Flint*—Ruthin, Aled, St. Asaph, Holywell and Hawarden RD's, Denbigh, Ruthin, Mold, Flint, Connah's Quay.
We. *Anglesey.*
Wf. *North coast*—Ogwen RD, Caernarvon, Bangor, Bethesda, Llanfairfechan, Penmaenmawr, Conway, Llandudno, Colwyn Bay, Abergele, Rhyl, Prestatyn.
Cg. *Chester* area—Chester and Tarvin RD's, Hoole.
Ch. *Runcorn* area—Runcorn RD, Runcorn, Lymm.
Ci. *Wirral* (*Mersey*)—Bebbington, Ellesmere Port.
Cj. *Wirral* (*Dee*)—Hoylake, Wirral, Neston.
Dk. *Cullompton.*
Dl. *Ottery St. Mary.*

*Comparison of Stomach Cancer Soils With Controls*

Table I shows that in every one of the 12 districts the mean logarithm of the zinc/copper ratios was greater for soils where stomach cancer had occurred (SC) than for the control group where there had been a death from a non-malignant cause (NM). When the 6 Welsh districts are combined by weighting each figure for SC and NM groups alike by the total soils from the district (SC + NM) the standardised means are 1·5329 for stomach cancer and 1·2821 for noncancer, the excess of 0·2508 being 3·9 times its standard error. When the 4 Cheshire districts are combined the standardised means are 1·3444 and 1·1369 respectively, the excess of 0·2075 being 2·5 times its standard error; and the Devon districts together show an excess of 0·1727 for the stomach cancer soils.

The alternative measure of the ratio, namely the number of soils in which the zinc content exceeded 30 times the copper content by weight, expressed as a percentage of the total soils in the group, shows an excess for stomach cancer over the control group in 11 out of 12 districts, and the standardised indices are 53·8 compared with 32·3 per cent in Wales, 44·3 compared with 27·1 in Cheshire and 88·6 compared with 76·8 in Devon.

As pointed out in previous papers organic carbon shows an excess for stomach cancer in all three of these areas when taken as a whole but in several districts, notably Lleyn and Wrexham, this is not seen. There are no appreciable correlations between log R and organic carbon in soils from the Welsh area ($r = 0{\cdot}095$ for NM and $r = 0{\cdot}039$ for SC) but in Cheshire there is a positive association for NM ($r = 0{\cdot}444$) though not for SC soils ($r = 0{\cdot}073$).

Since a chemical method for zinc determination replaced the spectrographic method in the course of this study of soils from Wales and Cheshire it seemed possible that some kind of bias might have resulted from this and zinc analyses by both methods were therefore carried out of 98 soils to ascertain whether there was any consistent difference between the results. The mean value according to the spectrographic method was 72·5 micrograms per gram, and by the chemical method it was 71·1, the difference being negligible. Individual samples showed considerable differences in some cases, as might be expected since different fragments of the sample were being compared, but it is evident that the discrepancies were not due to any consistent effect of the technique. The 350 soils from Wales and Cheshire in Table I comprised 74 analysed both ways, 175 by spectrography only and 105 by chemical method only. The chemical result was used in all cases where both methods had been used, but no appreciable change in the summarised mean values occurs if the spectrographic figures are used instead.

Another factor which might affect the zinc/copper ratio is the length of time during which the garden had been under cultivation, depending upon the age of the house which in turn would set limits to the number of years anyone could have lived in it. In order to investigate possible effects of this on Table I the data there used have been divided in Table II into residence durations of 10–19, 20–39 and 40 years or more; and three further groups have been examined, namely durations of 0–1 and 5–9 years and residual cases where the length of residence could not be ascertained. The mean values of log R in Wales and Cheshire have been standardised to the same distribution by districts as was used in Table I, and the comparison between stomach cancer and control groups is depicted in Fig. 2. The groups with unknown duration are there indicated on the supposition that they had the same mean duration as all the known cases.

The non-cancer group shows a slight tendency for the zinc/copper ratio to fall after 5–9 years and then to rise again after 10–19 years and the latter effect is more pronounced in Cheshire. Stomach cancer showed no tendency to occur where the soil had a higher ratio provided that the resident had lived less than 10 years in the house, but comparison with the control groups shows that there was such a tendency if the duration of residence had been 10–19, 20–39 or over 40 years, the excess in log R for the stomach group being about 0·25 to 0·3 for each of those

Table I.—*Comparison in 12 Districts of Zinc/Copper Ratio (R) in Garden Soils From Houses Where a Death Had Occurred From Stomach Cancer (SC) or a Non-malignant Cause (NM) After 10 or More Years Residence, Showing Also Average Amounts of Organic Carbon*

| | District | Cause of death | Total No. | Average content: Org. carb. | Zinc μg./g. | Copper μg./g. | Per cent R>30 | Logarithm of ratio: Mean value | (SC–NM) difference |
|---|---|---|---|---|---|---|---|---|---|
| Wa | Lleyn area | SC | 26 | 3·11 | 51·2 | 1·20 | 65 | 1·5581 | 0·6562 |
| | | NM | 13 | 3·66 | 20·1 | 2·46 | 8 | 0·9019 | |
| Wb | Snowdonia, Merioneth, | SC | 34 | 4·29 | 69·5 | 2·80 | 35 | 1·3306 | 0·1699 |
| | Denbigh moors | NM | 8 | 3·29 | 29·4 | 2·68 | 12 | 1·1607 | |
| Wc | Wrexham area (coal | SC | 19 | 3·01 | 142·7 | 2·19 | 79 | 1·7352 | 0·0814 |
| | mining) | NM | 9 | 3·61 | 198·9 | 3·17 | 56 | 1·6538 | |
| Wd | Clwyd and Flint (ex- | SC | 22 | 3·33 | 86·5 | 1·98 | 59 | 1·6503 | 0·0488 |
| | cept coast) | NM | 9 | 3·33 | 70·2 | 1·48 | 56 | 1·6015 | |
| We | Anglesey | SC | 26 | 3·86 | 43·8 | 1·21 | 62 | 1·5463 | 0·1917 |
| | | NM | 20 | 2·86 | 47·9 | 2·10 | 35 | 1·3556 | |
| Wf | North coast | SC | 40 | 3·15 | 129·5 | 3·91 | 48 | 1·4890 | 0·2741 |
| | | NM | 16 | 2·89 | 73·6 | 3·55 | 38 | 1·2149 | |
| Cg | Chester area | SC | 11 | 2·56 | 87·0 | 2·42 | 64 | 1·4472 | 0·0916 |
| | | NM | 14 | 2·36 | 66·4 | 2·46 | 50 | 1·3556 | |
| Ch | Runcorn area | SC | 11 | 2·23 | 43·1 | 4·62 | 0 | 0·9956 | 0·2533 |
| | | NM | 6 | 1·97 | 91·4 | 11·56 | 17 | 0·7423 | |
| Ci | Wirral (Mersey) | SC | 20 | 2·01 | 63·2 | 2·86 | 35 | 1·3103 | 0·3050 |
| | | NM | 15 | 2·04 | 65·2 | 4·74 | 20 | 1·0053 | |
| Cj | Wirral (Dee) | SC | 11 | 2·02 | 68·6 | 1·91 | 64 | 1·4913 | 0·1649 |
| | | NM | 20 | 2·15 | 43·4 | 1·90 | 30 | 1·3254 | |
| Dk | Cullompton | SC* | 31 | 3·49 | 176·9 | 2·67 | 90 | 1·7229 | 0·1509 |
| | | NM* | 24 | 3·22 | 118·9 | 2·62 | 75 | 1·5720 | |
| Dl | Ottery St. Mary | SC* | 7 | 3·29 | 306·6 | 3·39 | 86 | 1·8928 | 0·2116 |
| | | NM* | 24 | 2·82 | 120·0 | 1·95 | 80 | 1·6812 | |
| | | | | Standardised to same area distribution for SC, NM | | | | | |
| W | North Wales | SC | 167 | 3·46 | 86·2 | 2·32 | 53·8 | 1·5329 | 0·2508 |
| | | NM | 75 | 3·22 | 66·8 | 2·66 | 32·3 | 1·2821 | ±0·0649 |
| C | Cheshire areas | SC | 53 | 2·18 | 67·1 | 2·76 | 44·3 | 1·3444 | 0·2075 |
| | | NM | 55 | 2·04 | 62·4 | 4·47 | 27·1 | 1·1369 | ±0·0812 |
| D | Devon areas | SC* | 38 | 3·42 | 224·8 | 2·93 | 88·6 | 1·7841 | 0·1727 |
| | | NM* | 48 | 3·08 | 119·3 | 2·38 | 76·8 | 1·6114 | |

* Includes cases where the duration of residence was unknown.

Table II.—*Comparison Between Standardised Mean Logarithms of Zinc/Copper Ratio in Stomach Cancer and Control Groups of Garden Soils for Different Durations of Residence*

| Duration of residence | North Wales: Non-cancer (NM) No. | Mean | Stomach (SC) No. | Mean | Difference SC—MN | Cheshire: Non-cancer (NM) No. | Mean | Stomach (SC) No. | Mean | Difference SC—MN |
|---|---|---|---|---|---|---|---|---|---|---|
| 0–1 years | 22 | 1·4115 | .. | .. | .. | .. | .. | .. | .. | .. |
| 5–9 years | 16 | 1·4371 | 27 | 1·4246 | —0·0125 | 10 | 1·1138 | 13 | 1·1186 | +0·0048 |
| 10–19 years | 17 | 1·2203 | 57 | 1·5272 | +0·3069 | 23 | 1·0364 | 32 | 1·2985 | +0·2621 |
| 20–39 years | 32 | 1·2662 | 81 | 1·5131 | +0·2469 | 27 | 1·2603 | 19 | 1·4955 | +0·2352 |
| 40 or more | 24 | 1·2581 | 25 | 1·5593 | +0·3013 | 5 | 1·3533 | * | .. | .. |
| Not known | 45 | 1.3602 | 18 | 1·4580 | +0·0978 | 55 | 1·2151 | 16 | 1·3098 | +0·0947 |

* 2 soils only in this group.

FIG. 2.—Ratio of zinc to copper in garden soils related to residence duration.

Non-cancer ————

Stomach cancer - - - -

Other cancer ×

Unknown duration ○

durations. The groups with duration not known, whose average residence time was probably around 20 years, showed mean values of log R and differences between SC and NM groups which are consistent with better defined data.

*Comparison of Non-gastric Cancer Soils With Controls*

Data from 115 garden soils, 81 in Wales and 34 in Cheshire, taken at houses where a death from non-gastric cancer had just occurred were available for comparison with the non-cancer controls. Table III shows the mean values of the logarithm of the zinc/copper ratio, standardised for district distribution by the same weights as in previous tables, distinguishing residence durations of 5–9 years, 10 years or more and unknown. At over 10 years the non-gastric cancer soils had lower levels of log R than the controls, showing in Wales a small deficiency of 0·0461 compared with an excess of 0·2508 in Table I for stomach, and in Cheshire a deficiency of 0·0773 compared with an excess of 0·2075 for stomach. In the small groups with duration not known no significant differences are apparent.

Where the death had occurred after 5–9 years of residence no differences from the controls were found for stomach cancer (Table II and Fig. 2) but for other

TABLE III.—*Comparison Between Standardised Mean Logarithms of Zinc/Copper Ratio in Non-gastric Cancer and Control Groups of Garden Soils by Duration of Residence*

| Duration of residence | North Wales: Non-cancer (NM) No. | North Wales: Non-cancer (NM) Mean | North Wales: Non-gastric cancer No. | North Wales: Non-gastric cancer Mean | Cheshire: Non-cancer (NM) No. | Cheshire: Non-cancer (NM) Mean | Cheshire: Non-gastric cancer No. | Cheshire: Non-gastric cancer Mean |
|---|---|---|---|---|---|---|---|---|
| 5–9 years | 16 | 1·4371 | 41 | 1·4793 | 10 | 1·1138 | 17 | 1·3018 |
| 10 or more | 75 | 1·2533 | 32 | 1·2072 | 55 | 1·1751 | 9 | 1·0978 |
| Not known | 45 | 1·3602 | 8 | 1·3949 | 55 | 1·2151 | 8 | 1·2964 |

cancer there is a slight excess of 0·0422 in Wales and a larger excess of 0·1880 in Cheshire which is of doubtful significance. Analysis of these short duration groups according to the organ affected by cancer shows that the excess was appreciable only for cancers of the intestine (including rectum) for which the all-areas standardised value of log R for 25 soils was 1·5517 compared with 1·3634 for 33 cancers of the lung, breast and other organs and 1·3403 for 26 controls. This apparent excess in the zinc/copper ratio in soils associated with intestinal cancer after durations of residence between 5 and 10 years, but not after 10 years or more, might possibly be of significance but this cannot be decided unless larger numbers of such soils are obtained for analysis.

*Soil From Vegetable Gardens*

Samples were taken from the vegetable garden where such existed and of the 242 soils in North Wales detailed in Table I 131 or 54 per cent were so derived, and of the 108 in Cheshire 47 or 44 per cent, the remainder being taken from gardens where vegetables were not grown. The proportions over the whole area were about the same in the stomach cancer groups as in the controls. Table IV shows the mean organic carbon, zinc, copper and log R values for the SC and NM groups, distinguishing the vegetable garden soils from the rest and dividing the region into two parts of North Wales and two of Cheshire. All figures are standardised to correct for differences in detailed district distribution by using the same weights as in Table I.

The first area, Wa–b, comprising Snowdonia, Lleyn peninsula, Merionethshire and Denbigh moors, mostly mountainous, has an incidence of stomach cancer in men about 80 per cent above that in England, and in the vegetable gardens organic carbon shows a higher average content and higher SC/NM ratio than in the other areas. This is true also of the relative stomach cancer excess of zinc and of the corresponding deficiency of copper in the SC soils. The contrast between the values of log R in the SC and NM groups is very pronounced in this high mortality area, amounting to 0·582 in absolute and 63 per cent in relative terms in vegetable garden soils but only to about 0·2 and 19 per cent in other soils.

The second area, Wc–f, comprising the lower lying parts of North Wales to the north and east of Wa–b, has an incidence of stomach cancer in men about 33 per cent above that in England, and in the vegetable garden soils organic carbon shows an average content and SC/NM ratio intermediate between the values in Wa–b and the Cheshire areas where mortality from stomach cancer in not abnormal. This is true also of the relative zinc excess and copper deficiency in stomach

TABLE IV.—*Comparison Between Standardised Mean Indices of Organic Carbon, Zinc, Copper and Zinc/Copper Ratio in Stomach Cancer (SC) and Control (NM) Groups of Soils taken from Gardens Producing and not Producing Vegetables*

| District group | Subgroup | Number | Organic carbon | Zinc μg./g. | Copper μg./g. | Log zinc/Copper Mean | Log zinc/Copper SC—NM |
|---|---|---|---|---|---|---|---|
| | | | | Vegetable garden | | | |
| W a–b | NM | 10 | 3·17 | 23·3 | 2·81 | 0·9296 | |
| | SC | 40 | 3·93 | 66·4 | 1·63 | 1·5114 | +0·5818 |
| W c–f | NM | 29 | 2·90 | 62·9 | 2·15 | 1·3910 | |
| | SC | 52 | 3·43 | 83·2 | 1·80 | 1·6252 | +0·2342 |
| C g–h | NM | 12 | 2·23 | 58·5 | 2·66 | 1·2944 | |
| | SC | 13 | 2·34 | 54·0 | 2·31 | 1·3544 | +0·0600 |
| C i–j | NM | 13 | 2·27 | 53·7 | 2·34 | 1·3517 | |
| | SC | 9 | 1·98 | 37·9 | 2·06 | 1·2232 | −0·1285 |
| | | | | Other part of garden | | | |
| W a–b | NM | 11 | 2·97 | 27·9 | 2·31 | 1·0650 | |
| | SC | 20 | 3·63 | 52·0 | 3·06 | 1·2646 | +0·1996 |
| W c–f | NM | 25 | 3·28 | 109·3 | 3·14 | 1·3829 | |
| | SC | 55 | 3·19 | 120·4 | 3·68 | 1·5181 | +0·1428 |
| C g–h | NM | 8 | 2·27 | 84·5 | 9·24 | 0·9874 | |
| | SC | 9 | 2·47 | 81·0 | 6·25 | 1·0290 | +0·0416 |
| C i–j | NM | 22 | 2·00 | 51·8 | 3·57 | 1·0914 | |
| | SC | 22 | 2·05 | 76·2 | 2·66 | 1·4314 | +0·3400 |
| | | | Stomach cancer per cent of control (vegetable gardens) | | | | |
| W a–b | 100SC/NM | (181)* | 123 | 285 | 58 | 163 | .. |
| W c–f | ,, | (133)* | 118 | 132 | 84 | 119 | .. |
| C g–h | ,, | (105)* | 105 | 92 | 87 | 105 | .. |
| C i–j | ,, | ( 90)* | 87 | 71 | 88 | 90 | .. |

* These figures are the standardised mortality ratios for males in 1947–54 for cancer of the stomach (England and Wales = 100).

cancer compared with controls of the vegetable garden soils, and it applies to the absolute and relative excess in the mean value of log R, amounting to 0·234 and 19 per cent. In the other soils of this area both the SC and NM groups had high levels of zinc and of the zinc/copper ratio but the SC excess in log R was less than in the vegetable garden series, being only 0·143 and 10 per cent.

The third area, Cg–h, comprising Cheshire districts between the Welsh border and the Mersey apart from the Wirral, has a stomach cancer incidence in men about 5 per cent above the English average and organic carbon in the garden soils averaged less than in the Welsh areas with only a slight excess in the stomach cancer group. The differences between SC and NM groups in the values of log R were negligible in this area, although the copper level was high in the small group of other garden soils.

The fourth area, Ci–j, comprising the Wirral peninsula, has a stomach cancer incidence in men about 10 per cent below the English average and organic carbon levels were relatively low with no excess in the SC group of vegetable garden soils, and no excess either for log R. Considerable excess in log R was apparent however in the stomach cancer group of soils not taken from vegetable patches, amounting to 0·340 in absolute and 31 per cent in relative terms.

Considering that the four areas had mortality indices for cancer of the stomach in men of 181, 133, 105 and 90 respectively, the progression of the figures at the foot of Table IV for organic carbon, zinc, copper and the zinc/copper ratio in soils taken from vegetable gardens suggests that the explanation might lie in some common vegetable grown in gardens for domestic use, for no such correspondence for zinc, copper or log R is apparent for other garden soils. Alternatively actual contact with soil might account for it since soil is handled more in the course of cultivating and gathering vegetables such as potato than in looking after other parts of the garden.

### *Search for an Explanation*

It was shown experimentally by Howell (1958) that copper acetate when added to the diet had a strong retarding effect on hepatic tumour development in rats treated with *p*-dimethylamino-azobenzene (DMAB). This had been indicated by previous experiments and has received further confirmation by Fare and Woodhouse (1963). It is not possible at present to formulate any hypothesis as to how copper and zinc may be concerned in the genesis of neoplasms. Carcinogenesis is more complex than it was thought to be at one time, and can involve a number of elements such as chromium, arsenic, cobalt, nickel and beryllium as well as carbohydrates, hormones and viruses. Enzyme activity is essential in any organic change. The effects of trace-element imbalance may be to overemphasise one enzyme activity at the expense of another, different enzymes having different metals associated with them, or the enzyme may become associated with the " wrong " metal, leading to complete misfunction. This second possipility is unlikely in the case of copper and zinc.

The most that epidemiological studies can do is to reveal correlations between environmental or social factors and cancer incidence which may suggest lines of experimental work or means of prevention. It has already been shown that farmers and quarry workers in North Wales have higher death rates from stomach cancer than men in other occupations (Stocks, 1961), and that miners in Welsh coalfields show a greater excess of stomach cancer compared with other men in the same districts than is the case in other coalfields (Stocks, 1962), and these facts support the hypothesis that the high stomach cancer rates in most of Wales are connected with some peculiarity of the soil. The link might be by contamination of food or through home-grown vegetables, and to investigate the latter possibility potatoes were chosen for analysis simply because of convenience of their acquisition and handling. The potatoes were peeled and dried in thin sections, and these were milled and samples taken from the material for analysis of copper and zinc to determine whether the amounts present were related to those in the soil. Some pilot tests were made before standardising the technique.

A field study of farms was then carried out during 1961 in the county of Anglesey with the collaboration of Dr. G. Wynne Griffith who at that time was County Medical Officer of Health. Twelve parishes were chosen out of the 62 in the county, using female mortality from stomach cancer in 1948–60 as a criterion of selection, one group consisting of the 6 parishes having the lowest death rates and the other group consisting of the 6 with highest death rates. Four farms were visited in each parish and from each farm two samples of soil and of potato grown upon them were taken during the autumn, giving 48 matched pairs of soil and potato in each group. These were analysed for zinc and copper by chemical methods, and since no significant differences appeared between the two group averages the results for the whole series of 96 are shown in Table V.

TABLE V.—*Zinc and Copper in Farm Soils and in Potatoes Grown Upon them, With Comparative Data for Garden Soils in the Same County*

| Source of sample | Number of samples | Zinc µg./g. mean (z) | Copper µg./g. mean (c) | Zinc/copper ratio: Between means z/c | Zinc/copper ratio: Mean of log R |
|---|---|---|---|---|---|
| Potato (48 farms) | 96 | 14·0 | 4·97 | 2·8 | 0·454 |
| Farm soil (same) | 96 | 5·07 | 1·02 | 5·0 | 0·723 |
| Garden soils NM | 20 | 47·9 | 2·86 | 16·7 | 1·356 |
| in county* SC | 26 | 43·8 | 1·21 | 36·2 | 1·546 |

* After at least 10 years of occupation of house (Table I, We).

The farm soils from Anglesey contained much less zinc and rather less copper on the average than garden soils and their zinc/copper ratio was also lower, a finding to be expected in view of the differences in previous cultivation of fields compared with gardens. Potatoes had a higher content of each element per unit weight than the farm soils in which they were grown, and the average ratio of zinc to copper was about 3 in potatoes compared with 5 in the soils. The amount of copper in potato was weakly correlated with the amount of copper in the soil ($r = + 0{\cdot}200$ significant at 0·05 level), but not with the amount of zinc in the soil ($r = - 0{\cdot}043$). The amount of zinc in potatoes was not significantly correlated with either zinc or copper in the soil, but the zinc/copper ratios in potato and soil were intercorrelated to the extent of $r = + 0{\cdot}346$. It seems likely that this particular chemical balance in garden soils would tend to be reflected in potatoes grown in those gardens since this is found to be the case on farms.

If this subject is to be pursued further it would seem advisable (i) to compare potatoes grown on North Western and Fen soils with those grown on chalk soils, (ii) to determine directly whether zinc/copper ratios in potatoes grown in gardens at houses where stomach cancer has occurred tend to differ from those at other houses, analysing the soils at the same time, and (iii) to examine the influence of zinc and copper with the organic matter of soils (humic substances) on the development of stomach cancer in small animals.

### SUMMARY

Measurements of the zinc/copper ratio in some 750 soil samples from gardens in North Wales, Cheshire and two localities in Devonshire showed that in every one of 12 districts the average logarithm of this ratio was higher in gardens at houses where a person had just died after 10 or more years of residence of cancer of the stomach than it was at houses where a person had died similarly of a non-malignant cause. This effect was more pronounced and consistent in the soils taken from vegetable gardens than from gardens where no vegetables were grown, and it was not found where the duration of residence before death had been less than 10 years.

Similar comparison of gardens associated with other forms of cancer showed no differences from controls when the duration of residence had been 10 or more years, but a doubtful excess in the mean ratio appeared for intestinal cancers where the duration had been 5 to 9 years.

In potatoes grown on farm soils the zinc/copper ratio was positively correlated with that in the soil although the zinc content showed no such association.

Part of the expense of this investigation was defrayed by the British Empire Cancer Campaign for Research.

### REFERENCES

ABBOTT, D. C. AND POLHILL, R. D. A.—(1954) *Analyst*, **79**, 547.
FARE, G. AND WOODHOUSE, D. L.—(1963) *Brit. J. Cancer*, **17**, 512.
HOWELL, J. S.—(1958) *Ibid.*, **12**, 594.
NORDLING, C. O.—(1954) *Acta genet.*, **5**, 93.
SANDELL, E. B.—(1950) 'Colorimetric Determination of Metals.' 2nd Edition New York. (Interscience).
STOCKS, P.—(1953) *Brit. J. Cancer*, **7**, 40.—(1957) *Rep. Brit. Emp. Cancer Campgn*, **35**, Supplement pp. 111–113.—(1961) *Brit. J. Cancer*, **15**, 701.—(1962) *Ibid.*, **16**, 592.
*Idem* AND DAVIES, R. I.—(1960) *Ibid.*, **14**, 8.—(1961) *Rep. Brit. Emp. Cancer Campgn.*, **39**, 380.